# The Role of Histone Acetyltransferases and Histone Deacetylases in Photoreceptor Differentiation and Degeneration

**DOI:** 10.7150/ijms.43140

**Published:** 2020-05-23

**Authors:** Meng Zhao, Ye Tao, Guang-Hua Peng

**Affiliations:** 1 Laboratory of Visual Cell Differentiation and Regulation, Basic Medical College, Zhengzhou University, Zhengzhou 450001, China.; 2 Department of Pathophysiology, Basic Medical College, Zhengzhou University, Zhengzhou 450001, China.; 3 Department of Physiology, Basic Medical College, Zhengzhou University, Zhengzhou 450001, China.

**Keywords:** HDAC, HAT, photoreceptor, differentiation, degeneration

## Abstract

Photoreceptors are critical components of the retina and play a role in the first step of the conversion of light to electrical signals. The differentiation and degeneration of photoreceptors are regulated by specific genes and proteins. With the development of epigenetic approaches, scientists have discovered that histone modifications, such as acetylation, methylation, ubiquitylation, and phosphorylation, may modulate the processes of photoreceptor differentiation and degeneration. Histone acetylation is regulated by two opposing classes of enzymes, namely, histone acetyltransferases (HATs) and histone deacetylases (HDACs), which add and remove acetyl groups to and from target histones, respectively, causing changes in transcriptional activity. Herein, we review the effects of HATs and HDACs on the differentiation and degeneration of photoreceptors and discuss the underlying mechanisms of these effects.

## Introduction

The retina originates from the central nervous system and is defined as the “window to the brain.” The retina contains five major types of neuronal cells (photoreceptors, horizontal cells, bipolar cells, amacrine cells, and ganglion cells), as well as Müller glia cells, astrocytes, and microglia. In mammals, these cells are distributed throughout five laminar retinal layers, namely, the outer nuclear layer (ONL), outer plexiform layer (OPL), inner nuclear layer, inner plexiform layer (IPL), and ganglion cell layer^1^, ^2^. Photoreceptors, situated in the ONL and OPL of the vertebrate retina, can convert light into a neuronal signal, which is recognized as the beginning of visual processing. Photoreceptors comprise two types of neuronal cells, i.e., rods, which are responsible for dim light and night vision, and cones, which are responsible for daylight vision. Retinal rods are present in much larger numbers than cones. The nuclei of these cells are located in the ONL, and cone nuclei are distinctly larger and less condensed than rod nuclei^3^. Rod and cone photoreceptors establish synapses with bipolar cells in the OPL, while horizontal cells regulate synapse transmission. Cone bipolar cells interact with retinal ganglion cells and amacrine cells in the IPL. Amacrine cells are linked to ganglion cells, which are output neurons in the retina, projecting neural signals onto visual centers in the brain cortex^2^.

The transcription and translation processes in cone and rod photoreceptor cells are regulated by multiple transcriptional factors, such as the neural retina leucine zipper (NRL) protein, cone-rod homeobox (CRX) protein, orthodenticle homolog 2 (OTX2), nuclear receptor subfamily 2 group E member 3 (NR2E3) protein, and the retinal homeobox (RAX) protein; they also involve many pathways, such as the Sonic hedgehog (Shh), Notch, and thyroid hormone pathways^4^-^8^. Although the exact role of epigenetics in photoreceptor cell development and degeneration is not well known, epigenetic studies may be of great importance^9^. Epigenetics is the study of mitotically and/or meiotically heritable changes in gene function, which do not involve changes in the DNA sequence^10^. Posttranslational modifications of histone proteins, such as acetylation, methylation, ubiquitylation, and phosphorylation, can alter the structure of the chromatin around human genes. In general, according to its transcriptional state, the chromatin can be divided into actively transcribed euchromatin and transcriptionally inactive heterochromatin^11^. Histone acetylation, which mainly occurs at lysine residues in the N-terminal tail of the core histones H2A, H2B, H3, and H4, is associated with various physiological and pathological processes through changes in the chromatin structure and gene expression levels^12^. The homeostasis of the acetylation state is regulated by two classes of antagonizing histone-modifying enzymes, histone acetyltransferases (HATs) and histone deacetylases (HDACs). These histone-modifying enzymes can add or remove acetyl groups to or from target histones, respectively, leading to an increase or decrease in transcriptional activity and thus regulating the expression of target genes^13^, ^14^. Emerging studies show that HATs and HDACs play critical roles in the differentiation and degeneration of photoreceptors and indicate that HDAC inhibitors may have a therapeutic potential in some retinopathies.

Therefore, this review discusses the roles of HATs and HDACs in photoreceptor differentiation and degeneration, reviews studies on the underlying mechanisms of these effects, and highlights the therapeutic potential of targeting HATs and HDACs for the treatment of ophthalmic diseases caused by photoreceptor degeneration.

## HATs and HDACs

Mammalian HATs are divided into three families. The Gnat5 family, whose main substrates are histone proteins, includes Gcn5, PCAF, Hat1, Elp3, and Hpa2; the MYST family, whose main substrate is the H4 protein, comprises Esa1, MOF, Sas2, Sas3, MORF, Tip60, and Hbo1; and the p300/CBP family comprises p300 and CBP, which act on H3 and H4^15^. Recent research has revealed that HATs contribute to nucleosome assembly, DNA repair and transcription, and protein deposition^12^, ^16^ (Table 1).

In total, 18 human HDACs have been identified so far. Based on developmental genetics and sequence similarity with the yeast proteins RPD3, HDA1, SIR2, and HOS3^17^, human HDACs have been grouped into the following four classes: class I (HDAC1, HDAC2, HDAC3, and HDAC8); class II, which is further divided into two subclasses: IIa (HDAC4, HDAC5, HDAC7, and HDAC9) and IIb (HDAC6 and HDAC10); class III (SIRT1, SIRT2, SIRT3, SIRT4, SIRT5, SIRT6, and SIRT7); and class IV (HDAC11)^18^, ^19^. Class I, II, and IV enzymes are Zn^+^-dependent protein deacetylases, while class III enzymes are NAD^+^-dependent protein deacetylases. Class I HDACs are mainly located in the nucleus and contribute to cell development and differentiation, especially in neurogenesis. Class II HDACs are present both in the cytoplasm and nucleus and collaborate with class I proteins, whereas class III HDACs are independent and located in the nucleus, mitochondria, or cytoplasm^20^, ^21^ (Table 2).

## HDACs, HATs, and differentiation of photoreceptor cells

The differentiation of photoreceptor cells starts from retinal progenitor cells. First, multipotent retinal progenitor cells proliferate into retinal progenitor cells. Next, progenitor genes are gradually silenced, and photoreceptor-specific genes are expressed as photoreceptor precursors, which differentiate into mature photoreceptor cells. Finally, synapses are formed through axonal growth and outer segment formation^22^. In humans, photoreceptors are generated before birth, and cone receptor formation occurs 2 weeks before rod receptor formation^23^.

Recent studies have revealed underlying regulatory mechanisms of the HAT and HDAC effects on gene expression and photoreceptor differentiation. c-Jun N-terminal kinase 1 (JNK1) can directly interact with NRL, phosphorylating its serine-50 residue and enhancing NRL activity. Phosphorylated NRL binds to the rhodopsin and Ppp2r5c promoters, activating their transcription through the recruitment of the HIV Tat-interacting protein (Tip60) to promote histone H3/H4 acetylation^24^. These data suggest that the cooperation among NRL, JNK1, and Tip60 plays a regulatory role in photoreceptor differentiation.

Another study illustrated the role of HATs in photoreceptor differentiation during retinal development by assessing CRX expression. One way of activating the transcription of this protein is to recruit the transcriptional coactivators p300 and CBP to acetylate promoter-bound histones. A conditional knockdown of p300 and CBP in differentiating rods or cones reduced the histone H3/H4 acetylation and consequently decreased the transcription levels of photoreceptor-specific genes, disrupting the function and structure of these cells^25^.

From postnatal day 2 to 10, the inhibition of class I and II HDACs with trichostatin A (TSA) was shown to cause the numbers of rod photoreceptors to drop; it also caused transcription factors that are essential for rod differentiation, such as OTX2, CRX, and NRL, as well as NeuroD1 expression (necessary for rod photoreceptor survival) to decrease within 3 h^26^. These results indicated that HDACs could promote the differentiation of rod photoreceptors and played a key role in deciding the fate of photoreceptor progenitors, although further efforts are still needed to elucidate the exact role of each HDAC. Using a specific HDAC1 inhibitor in mice, Ferreira et al.^27^ demonstrated that the transcription levels of *Vsx2*, *Hes1,* and other progenitor-specific genes remained stable because their promoters were acetylated. By contrast, the expression levels of *Nrl*, *Rho,* and other rod-specific genes decreased because of a reduction in histone acetylation. These authors tested three histone sites and discovered that the acetylation of H3K9 and H4K12 increased, while that of H3K27 did not change upon HDAC1 inhibition. These findings suggest that HDAC1 is a key protein in the process leading a progenitor cell to form a terminally differentiated rod photoreceptor; however, HDAC3 did not show similar functions in the differentiation of rod photoreceptors after birth^27^.

Cell division is the basis of growth, development, and reproduction of individuals in multicellular organisms. The destiny of cells is related to the stage of the cell cycle in which they are found. Cells with differentiation potential stay in the G0 phase and reenter a new cell cycle to become differentiated when induced^28^, ^29^. The differentiation of retinal progenitor cells into retinal neurons is regulated during development by cell-cycle molecules. Therefore, it is essential to investigate the cell cycle of photoreceptor cells and the mechanism of its regulation by HATs and HDACs. Using *hdac1* mutant models to study the zebrafish retina, Stadler et al.^30^ found that HDAC1 was essential for the cell-cycle exit during retina differentiation, which was accompanied by a reduction in the cyclin D and E levels. Cyclins D and E are the drivers of cell-cycle progression, and their regulation is region and species specific. Cyclin D1 interacts with the *Notch1* gene, where it recruits the CBP HAT during mouse retinal development^31^. The retinoblastoma protein (Rb) can bind to the tumor suppressor protein E2F and form a cell-cycle regulator complex, which functions alongside HDACs^32^. These studies indicate that HDACs and HATs can affect the cell cycle of photoreceptors during their development; however, more in-depth research is still required in this field.

## Degeneration of photoreceptor cells

DNA sequences, transcription patterns, and translation must function in an error-free and coherent manner to maintain the normal function and homeostasis of photoreceptors. Therefore, gene mutations, transcriptional disorders, and microenvironmental changes can lead to photoreceptor loss or dysfunction. Generally, photoreceptor diseases can be classified as nature and nurture types. The best-studied primary inherited rod degenerative diseases, which are followed by cone degeneration, are retinitis pigmentosa (RP) and Leber congenital amaurosis (LCA)^33^. Primary inherited cone degenerative diseases include Stargardt's and Best's diseases, achromatopsia, and cone dystrophies. Cone dystrophies are caused by at least 27 gene mutations and can be affected by age-related macular degeneration (AMD) or diabetic retinopathy^34^.

LCA is a severe rod-cone dystrophy disease that can lead to blindness shortly after birth. Autosomal recessive inheritance is the main inheritance pattern in patients with LCA, and more than 20 related gene mutations have been identified to date^35^. RP is an ocular disease that causes the progressive death of photoreceptor cells is thought to be controlled by apoptosis^36^. The first RP-related gene mutation was reported in 1990, and more than 100 such gene mutations have been identified thus far^37^. The first general symptom of RP is night blindness, which is followed by a loss of central vision and eventually full blindness^38^. Different genotypes can result in the same phenotype, and, vice versa, one genotype may result in different phenotypes. Many factors are involved in photoreceptor degeneration. The DNA sequence, transcription, posttranscriptional modifications, translation, and posttranslational modifications are five main elements that can influence the function of the final protein; each element represents a different area of research. However, with the rapid development of epigenetics in recent years, researchers have gradually discovered that retinal degeneration is closely associated with epigenetic regulation^39^.

## Effects of HDACs and HATs on photoreceptor degeneration, underlying mechanisms of action, and potential therapies

Animal models are necessary for the study of retinal degenerative diseases, such as RP and AMD^40^. Cyclic nucleotide phosphodiesterase-6 (PDE6) is a key enzyme that regulates the intracellular levels of cyclic guanosine monophosphate (cGMP). A mutation in *PDE6* can lead to cGMP accumulation, which further results in a loss of photoreceptors^41^. Two known mutations in the loci of the PDE6β and PDE6c subunits are *rd1* (retinal degeneration 1) and *cpfl1* (cone photoreceptor function loss 1), which result in an early onset and fast progressive death of rods and cones, respectively^42^, ^43^. The pathogenesis of *rd1* is diverse, including endogenous molecular stimulation in photoreceptor cells and changes in the microenvironment that cause abnormal cell degeneration and death, such as calcium disorders in *rd1* mice^44^. The expression of HDACs in a degenerative retina has not yet been sufficiently investigated. The concentration of Sirt1, which is located in the nucleus, increases with photoreceptor degeneration, resulting in an anti-apoptosis protection of the retina, which is related to the DNA repair mechanism and homeostasis maintenance in *rd1* mice^45^.

To reveal the effect and determine the mechanism of action of HDACs on photoreceptor loss and to search for a therapeutic alternative, most studies repress specific HDAC proteins using gene knockdown or pharmacological drugs. The latter can be divided into selective and non-selective categories, depending on the targeting efficiency. TSA, a hydroxamic acid, is a selective HDAC inhibitor, specific for class I and II HDACs^46^. Vorinostat (SAHA), a hydroxamic acid derivative and selective HDAC inhibitor, was the first drug approved by the Food and Drug Administration for applications in cancer treatments^47^. A short-chain fatty acid, valproic acid (VPA), is also a selective HDAC inhibitor and is currently used as an anticonvulsant drug^48^. These three HDAC inhibitors are the most commonly used drugs in therapeutic studies of retinal diseases.

Among six studies of clinical treatment of RP with VPA, in which the time of administration varied from 4 to 12 months and the dosage was 400 or 500 mg/day, only one study, conducted in 2017^49^, showed negative results. In five other studies, the outcomes of the drug treatment in patients with RP were compared with those of the control treatment, as well as with the baseline conditions, and the results showed that VPA treatment improved the visual field and visual sensitivity and delayed vision loss (p<0.05)^50^-^54^. Although VPA had a positive impact on RP, some studies suggested that this treatment was not appropriate for all genotypes, and it was even prohibited in some cases of RP^55^, ^56^. Therefore, the confirmation of genetic mutations is required before VPA administration. In addition, the long-term efficacy, dosage, standardization, and safety of VPA should all be considered during a clinical trial. In a *cpfl1* retina, treatment with TSA could significantly improve cone survival, as well as the cone migration pattern, while SAHA could prevent rod death in *rd10* retinal explant cultures^34^, ^57^. HDACs were highly expressed in an *rd1* retinal explant culture, and injection of TSA resulted in a significant improvement of rod survival^58^. In the 661W cell line, treatment with TSA resulted in an increased binding of E2F-1 and p53 to the *Apaf-1* promoter, which in turn led to increased Apaf-1 expression and subsequent apoptosis of cone cells^59^. Additionally, VPA could protect photoreceptor cells from *N*-methyl-*N*-nitrosourea-induced apoptosis, not only through its anti-apoptotic effects but also through its ability to inhibit HSP70 degradation^60^.

The retina is an energy-consuming organ. Metabolic processes, such as glycometabolism and oxidative stress, are related to photoreceptor death, and HDACs play a regulatory role in this process. Acute hypoglycemia can impair human retinal photoreception, cause central vision injury, and eventually lead to blindness^61^. SIRT6 can control glucose homeostasis and maintain normal retinal function through the deacetylation of the H3K9 and H3K56 sites of multiple glycometabolic genes, such as *GLUT1* and *GRM6*^62^. SIRTs require NAD^+^ as a cofactor, which makes them highly sensitive to oxidative stress. One process of a pathological change in AMD involves the initiation of SIRT1 activation by hypoxia to enhance the expression of hypoxia-inducible factor 2α, which in turn activates the release of the vascular endothelial growth factor^63^. HDAC6 exists both in the nucleus and cytoplasm, and in the cytoplasm, it can act on its substrate protein, tubulin. Selective inhibition of HDAC6 with tubastatin A could protect photoreceptors from reactive oxygen species by enhancing the expression of HSP70, HSP25, and peroxiredoxin 1^64^. DNA microarray assays showed that genes related to retinal proliferation and oxidative stress were upregulated in *rd1* mice. The exact relationships between these genes and activities of individual HDACs have not yet been elucidated, although one study found an increase in class I and II HDACs before retinal degeneration^58^, ^65^. The overexpression of HDAC4 in electroporated *rd1* mouse retinas could prolong the photoreceptor survival in a way that might be related to hypoxia-inducible factor 1α activity in the cytoplasm^66^. These results seem to be different from previous data^34^, ^57^, ^67^, which showed that the inhibition of HDACs could protect photoreceptors from degeneration under certain conditions. One explanation may be that HDAC4 plays a neuroprotective role for photoreceptors when it is located in the cytoplasm, which indicates that its substrates are not histone proteins. Moreover, the abovementioned results may indicate that HDACs have multiple functions in photoreceptor degeneration; therefore, more studies are needed to elucidate the mechanisms underlying the role of HDACs in photoreceptor degeneration. A recent study^68^ suggested that a short N-terminal domain of HDAC4 plays a critical role in photoreceptor protection, thus confirming the abovementioned results.

Effective treatment has not been hitherto found for hereditary photoreceptor degeneration^69^, but two potential therapies are being developed. One involves gene therapy, and the other is replacement therapy, which involves the production of new photoreceptor cells from stem cells^70^, ^71^. To establish the regulatory role of epigenetic mechanisms, we focused on the process of differentiation of stem cells into photoreceptors. Depending on their origin, stem cells can be classified as exogenous, including mesenchymal, neural, embryonic, and induced pluripotent stem cells, and endogenous, including Müller glia, ciliary epithelial, and retinal pigment epithelial cells^72^. It will be a major focus in the future to determine ways to build and advocate for an ethical embryonic stem cell bank for the benefit of humanity. Recent studies have successfully derived rod photoreceptors from mouse and human embryonic stem cells^71^. Müller glia cells in zebrafish can transdifferentiate into photoreceptors, while those in mammals cannot spontaneously regenerate. It is important to note that epigenetic regulation, including histone acetylation, may act as a key stimulation switch in the trans-differentiation of Müller glia cells into photoreceptors 73. The success of photoreceptor regeneration is only the first step in replacement therapy, and successful cell transplantation to recipients remains a great challenge. Scientists are pursuing this goal by studying the right moment for transplantation, repression of the immune reaction after transplantation, and establishment of an appropriate growth microenvironment^74^-^76^. The exact modulatory effects of epigenetic factors, including histone acetylation, on the process of differentiation from stem cells to photoreceptors remain to be explored.

## Discussion

With the development of epigenetic approaches, the study of human diseases is no more limited to DNA sequencing. Histone acetylation, an epigenetic modification, mostly occurs at specific Lys residues in the N-terminal alkaline amino acid region of core histones. While HATs acetylate histones, which results in their dissociation from negatively charged DNA, a loosening of the chromatin structure, and the initiation of gene transcription, HDACs deacetylate histones, which results in their tight binding to negatively charged DNA, a dense chromatin packaging, and the suppression of gene transcription. The effect of HATs and HDACs on retinal degeneration has recently gained a significant research interest. In this review, we principally focused on the diseases caused by photoreceptor degeneration in an attempt to reveal the relationships among HATs, HDACs, and photoreceptor differentiation and degeneration.

The effects of HDACs on photoreceptors were the main focus of the studies reviewed, and the association between HATs and photoreceptor degeneration remains to be further explored. Most studies focused on general effects of HDACs on photoreceptor cells, while specific effects of individual HDACs remain to be elucidated. In most cases, studies used pharmacological HDAC inhibition to reveal the effects of HDACs, and the specificity of an HDAC inhibitor should be considered to establish the precise underlying mechanism of HDAC effects on photoreceptor differentiation and degeneration. To activate and repress the transcription of HATs and HDACs, they must form activator or repressor complexes with other proteins, such as transcription factors; therefore, exploring these complexes should be a key research focus in future studies. Moreover, multiple molecules and pathways participate in the differentiation and degeneration of photoreceptor cells and influence their physiological and pathological processes. Therefore, clarifying the roles of HATs and HDACs in photoreceptor differentiation and degeneration and constructing a complete reticular regulation map are necessary for the prevention of and development of treatment options for photoreceptor degeneration. The mechanisms of differentiation, degeneration, and redifferentiation of photoreceptors significantly vary among species. Research on exact differences in these mechanisms among species is another important area of focus to correlate the results obtained in different animal models with those obtained in humans. With the development of epigenetic high-throughput sequencing technology, more breakthroughs will be achieved in the elucidation of the effects of HATs and HDACs on photoreceptors. These enzymes may also represent promising targets for the treatment of ophthalmic diseases caused by photoreceptor degeneration.

## Figures and Tables

**Table 1:** HAT family members^77^-^93^

Class	Name	Histone target sites
Gcn5 family	Gcn5	H2BK11, H2BK16, H3K9, H3K14, H3K23, H3K36, H4K8, H4K16
	PCAF	H3K14, H4K8
	Hat1	H2AK7, H4K5, H4K8, H4K12
	Elp3	H3K14, H4K8
	Hpa2	H3K4, H3K14, H4K5, H4K12
MYST family	Esa1	H2AK4, H2BK16, H3K4, H3K14, H4K5, H4K8, H4K12, H4K16
	MOF	H4K16
	Sas2	H3K14, H4K16
	Sas3	H3K14, H3K23
	Tip60	H2AK5, H3K14, H4K5, H4K8, H4K12, H4K16
p300/CBP family	P300	H2AK5, H2BK5, H2BK12, H2BK15, H3K14, H3K18, H3K23, H3K27, H4K5, H4K8, H4K12
	CBP	H2AK5, H2BK12, H2BK15, H3K18, H3K23, H3K27

**Table 2:** HDAC family members^18^, ^94^, ^95^

Class	Subtype	Protein domain	Number of amino acids	Protein size (kDa)	Localization	Yeast homolog
Ⅰ	HDAC1	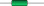	482	58	Nucleus	RPD3
	HDAC2	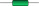	488	59	Nucleus	
	HDAC3	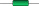	428	50	Nucleus, cytoplasm	
	HDAC8	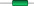	377	44	Nucleus	
Ⅱa	HDAC4	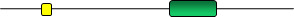	1,084	120	Nucleus, cytoplasm	Hda1
	HDAC5	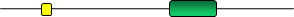	1,122	130	Nucleus, cytoplasm	
	HDAC7	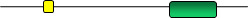	912	110	Nucleus, cytoplasm	
	HDAC9	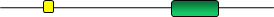	1,011	160	Nucleus, cytoplasm	
Ⅱb	HDAC6	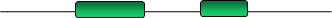	1,215	160	Nucleus, cytoplasm	
	HDAC10	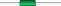	669	70	Nucleus, cytoplasm	
Ⅲ	Sirt1	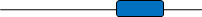	747	120	Nucleus	Sir2
	Sirt2	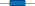	389	45	Cytoplasm	
	Sirt3	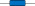	399	28	Mitochondria	
	Sirt4	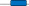	314	35	Mitochondria	
	Sirt5	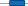	310	36	Mitochondria	
	Sirt6	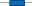	355	39	Nucleus	
	Sirt7	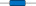	400	48	Nucleus	
Ⅳ	HDAC11	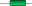	347	39	Nucleus	HOS3

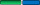
Catalytic domain 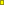
MEF-binding domain

## Abbreviations

HAT: histone acetyltransferase
HDAC: histone deacetylase
JNK1: c-Jun N-terminal kinase 1
NRL: neural retina leucine zipper
Tip60: HIV Tat-interacting protein
CRX: cone-rod homeobox
TSA: trichostatin A
PNR: photoreceptor cell-specific nuclear receptor
Rb: retinoblastoma protein
RP: retinitis pigmentosa
LCA: Leber congenital amaurosis
AMD: age-related macular degeneration
PDE6: cyclic nucleotide phosphodiesterase-6
cGMP: cyclic guanosine monophosphate
*rd1*: retinal degeneration 1
*cpfl1*: cone photoreceptor function loss 1
SAHA: vorinostat
VPA: valproic acid
ONL: outer nuclear layer
OPL: outer plexiform layer
IPL: inner plexiform layer
OTX2: orthodenticle homolog 2
*RAX*: retinal homeobox gene
Shh: Sonic hedgehog
Ret-CoR: photoreceptor cell-specific nuclear receptor co-repressor
